# Amino Acid Levels as Potential Biomarker of Elderly Patients with Dementia

**DOI:** 10.3390/brainsci10120914

**Published:** 2020-11-27

**Authors:** Edyta Socha, Piotr Kośliński, Marcin Koba, Katarzyna Mądra-Gackowska, Kornelia Kędziora-Kornatowska, Marcin Gackowski, Emilia Daghir-Wojtkowiak

**Affiliations:** 1 Department of Toxicology and Bromatology, Faculty of Pharmacy, Collegium Medicum of Nicolaus Copernicus University, 85-067 Bydgoszcz, Poland; edyta.siminska@gmail.com (E.S.); piotr.koslinski@cm.umk.pl (P.K.); marcin.gackowski@cm.umk.pl (M.G.); 2 Department of Geriatrics, Faculty of Health Sciences, Collegium Medicum of Nicolaus Copernicus University, 85-067 Bydgoszcz, Poland; katarzyna.madra@cm.umk.pl (K.M.-G.); kasiakor@interia.pl (K.K.-K.); 3 Department of Biopharmaceutics and Pharmacodynamics, Medical University of Gdańsk, 80-210 Gdańsk, Poland; emilia.daghir@gmail.com

**Keywords:** neurodegenerative diseases, mild dementia, moderate dementia, amino acids, arginine, serine, isoleucine

## Abstract

Dementia is a clinical syndrome characterized by cognitive impairment, in which there is disturbance of multiple higher cortical functions. The primary risk factor of dementia is old age, and due to significant changes in the worldwide demographic structure, the prevalence of cognitive impairment is increasing dramatically with aging populations in most countries. Alzheimer’s disease is the predominant and leading cause of dementia. The aim of this study was to evaluate the modifications of amino acids that characterize the initial stages of dementia to help our understanding of the complex and multifactorial pathogenesis of neurodegenerative disorders. A total of 123 participants were divided into two groups: healthy elderly subjects and patients with mild or moderate dementia. The results of this study indicate that the serum levels of three amino acids were changed significantly in patients with dementia, in relation to the subjects without dementia. In particular, we observed differences in concentrations for serine, arginine and isoleucine (all of them were significantly increased in patients with dementia, compared with the control group). Our results suggest that the metabolisms of some amino acids seem be changed in patients with dementia. We conclude that amino acid profiling might be helpful for the better understanding of biochemical and metabolic changes related to the pathogenesis and progression of dementia. However, considering the multifactorial, heterogenous and complex nature of this disease, validation with a greater study sample in further research is required.

## 1. Introduction

Dementia is a clinical syndrome characterized by cognitive impairment, in which we observe the disturbance of multiple higher cortical functions, including thinking, memory, orientation, calculation, comprehension, language, learning capability and judgment [1]. The primary risk factor of dementia is old age, and due to significant changes in the worldwide demographic structure, the prevalence of cognitive impairment is increasing dramatically with aging populations in most countries. Currently, neurodegenerative disorders are one of the greatest global challenges for medical and social care, due to an aging society and the rapidly increasing costs of dementia [2].

Dementia occurs in cerebrovascular disease, Parkinson’s disease and in other conditions affecting the brain, but indisputably, Alzheimer’s disease (AD) is the predominant and leading cause of dementia [1]. Based on the most recent available data, AD is estimated to be responsible for up to 80% of dementia cases. Therefore, the authors of this paper mainly refer to AD as a leading cause of dementia [3].

Research to date has tended to focus on the amyloid cascade hypothesis in the development of AD. However, increasing recent evidence suggests that this hypothesis indicating the role of amyloid-*β* (Aβ) protein aggregation in AD pathogenesis does not encapsulate the heterogenous and complex symptomology of AD [4,5]. Growing clinical evidence shows that AD-associated cognitive impairment is the outcome of an especially complex pathogenesis. In addition to the well-defined and known amyloid plaques and neurofibrillary tangles, the pathology of AD includes inflammation, considerable DNA damage, substantial neuronal loss, the initiation of apoptotic pathways, chronic oxidative stress, alteration of the energy metabolism, and extensive mitochondrial malfunction. Long-term failures to find effective anti-Tau or anti-amyloid therapy have led to the hypothesis that specific proteins are only confluent lesions and not the sole and ultimate causes of AD. Numerous recent papers present evidence pointing to severe metabolic dysfunction as a leading cause of AD. Current data show the causative relationship between obesity and AD [6,7]. Furthermore, numerous studies reported significant improvement in the cognitive, functional, and cellular statuses of AD patients who were treated with conventional medicines, used in treating atherosclerosis, diabetes, and other metabolic disorders [6,8,9]. Hence, the current vision of AD pathogenesis must reach beyond the confirmed regular information and look for alternative research approaches in order to develop novel diagnosis, preventive, and therapeutic methods. It is very important to quest for better understanding of the biochemical changes at different stages of the disease, which are an integral part of the complex and multifactorial pathogenesis of dementia.

It is essential to advance the understanding of early disease mechanisms and to identify early biomarkers and novel therapeutic targets. Currently available diagnostic biomarkers are limited to the measurements of the Aβ, tau, and p-tau levels in plasma and cerebrospinal fluid (CSF) that represent rather limited, hypothesis-driven biomarker development [4,6]. Furthermore, neuroimaging is very expensive and available only at specialized sites. Most health care systems in the world cannot afford using Positron Emission Tomography (PET) as a routine diagnostic tool for screenings of the very high number of patients from the risk group (elderly subjects in an aging society). The limitations in the routine use of CSF biomarkers include the lumbar puncture, which is invasive, time-consuming, and complicated for many clinics [10].

Taking into account the current state of dementia diagnostics, it is important to identify a putative biomarker. In recent years, there has been an increasing interest in such research, with the use of various easy-to-collect biological materials such as serum (e.g., our study) or saliva. It is interesting to note that several recent studies indicated the antioxidant properties of saliva are limited in patients with dementia, and salivary redox biomarkers may be used for differentiating the various stages of dementia. Despite the very interesting and promising results, there are some important limitations, such as factors affecting the quantity and quality of saliva (e.g., drugs, periodontal diseases, oral mucosa disorders, age, various systemic diseases, xenobiotics, dental materials, dental treatment, physical exercise, and diet). Unfortunately, a perfect dementia biomarker or diagnostics method still does not exist, and it is important to conduct new research and strive to improve and develop the diagnostics area [11,12]. Several recent studies have identified changes in the plasma amino acid (AA) profile in the material of patients with AD and other advanced stages of neurodegenerative diseases [13,14,15,16,17,18,19,20], but there is still insufficient data for this aspect that takes into account the earlier stages of dementia. Amino acids play essential roles in the regulation and control of pivotal functions of human organisms, including the central nervous system (CNS), where AAs act as regulators of the energy metabolism, neurotransmitters, and neuromodulators. Neurotransmission is a key function of AAs in the CNS. Neurotransmission amino acids are compounds that are synthesized and stored in neurons. They are released when the nerve impulse is conducted. Alterations in plasma amino acid profiles in neurodegenerative diseases may be influenced by neurodegeneration, received treatment, mitochondrial disfunction, or malabsorption in the gastroenteric tract [15,20].

Furthermore, amino acids are involved in energy production and nitrogenous waste production and elimination as well. The human body needs amino acids in large amounts for the synthesis of body proteins and other important nitrogen-containing compounds, such as peptide hormones, creatine, and some neurotransmitters. Hence, amino acids are a large and important part of the human diet. Processing these essential amino acids for energy requires the disposal of nitrogenous waste material, a process which occurs primarily in the liver and small intestine during the urea cycle. Disturbed amino acid and nitrogen metabolism is associated with neurological disorders and, in some cases, dementia. When neurons cannot catabolize glucose effectively, they may become reliant upon amino acid oxidation during energy production. If the mechanism used to metabolize amino acids becomes dysregulated, or if neuronal amino acids become depleted, the neurons may die. This might contribute to disease progression. In addition to these main functions, amino acids and their metabolic derivatives are involved in cell signaling and in many different metabolic pathways [4].

The aim of this study was to evaluate the modifications of amino acids that characterize the initial stages of dementia to help our understanding of the complex and multifactorial pathogenesis of dementia.

## 2. Materials and Methods

### 2.1. Subjects and Serum Samples

A total of 123 participants were recruited from the Department of Geriatrics, Collegium Medicum in Bydgoszcz, Nicolaus Copernicus University in Toruń. They were divided into two groups: 50 healthy elderly subjects with no dementia diagnosed (23 males, 27 females; mean age ± SD: 77.09 ± 7.08 years) and 73 patients with mild or moderate dementia (17 males, 56 females; mean age ± SD: 81.89 ± 6.38 years). Table 1 reports the clinical and demographic characteristics of the two groups of participants.

Investigators classified patients into two groups in accordance with the current clinical standard: a group of elderly patients without dementia and a group of elderly patients with dementia (from mild dementia to moderate dementia). Participants of the study were chosen from older adults, who were referred to a comprehensive geriatric assessment by a general practitioner, mainly because of persistent symptoms of memory loss reported over a six-month span. Patients were included in the study prior to a clinical diagnosis of the cause of dementia. They were admitted to the geriatric clinic for a comprehensive geriatric assessment to evaluate their cognitive status. Patients with causes of reversible dementia were excluded. Patients had laboratory tests ordered to rule out reversible causes of dementia (e.g., B12 deficiency or hypothyroidism). The International Statistical Classification of Diseases and Related Health Problems (ICD-10) criteria were adopted for the clinical diagnosis of dementia. Furthermore, diagnosis of dementia was based on a review of the patient’s medical history and symptoms via a physical examination and a brain scan, such as a CT or MRI scan. A mini-mental state examination (MMSE) and a clock-drawing test (CDR) were used to diagnose the severity of dementia. The mean score on the MMSE for healthy elderly subjects with no dementia diagnosed was 27 points. For patients with mild dementia, it was 21 points, and for patients with moderate dementia, it was 16 points.

The assignment to the groups was documented in a survey that was previously reviewed and approved by the ethics committee. This study was conducted with all approved international guidelines for human research and was in accordance with the Declaration of Helsinki. The ethics committee of Nicolaus Copernicus University in Toruń and Collegium Medicum in Bydgoszcz approved this study (consent number: KB 173/2018). Written, informed consent was required from the participants. An informed consent form, an investigator survey form, and a patient survey form were also reviewed and approved by the ethics committee.

Antecubital whole-blood samples were drawn from a peripheral vein in the morning hours (always between 6 and 7 a.m.). Overnight fasting and 15 min of rest before the blood test were obligatory. The needle was inserted into the vein, and the blood was withdrawn to vacuum tubes (the test’s 5 mL tube contained a clot activator and serum gel separator, and it had no anticoagulant). Blood collected in the tube was kept for 30 min. Serum from the blood after clotting was separated out and, after appropriate preparation and centrifuging, was frozen at −80 °C until analysis was performed.

### 2.2. Chemicals and Materials

A system consisting of an AccQ Fluor reagent kit (Waters, En Yvelines Cedex, France, amino acid standards (Waters), internal standard α-aminobutyric acid (Sigma Aldrich, Saint Louis, MO, USA), acetonitrile and methanol (Sigma Aldrich), and deionized water purified with Direct–QUV (Millipore, Molsheim, France) was used for all aqueous solutions.

### 2.3. Instrumentation

A Shimadzu high-performance liquid chromatography (HPLC) system, combined with a Diode-Array Detection (DAD) detector and an RF-20A XS fluorescence detector (Shimadzu, Kyoto, Japan) were used.

### 2.4. Chromatographic Method

Chromatographic analysis, which was aimed at measuring the amino acid concentrations in the samples, was carried out using high-performance liquid chromatography (HPLC) with fluorescence detection, using an AccQ Tag column (Waters). The final parameters of the chromatographic system consisted of a 150 mm × 3.9 mm chromatographic column by AccQ Tag (Waters), a fluorescence detection excitation/emission of 250/395, mobile phase A conducted with an AccQ Tag Eluent A (Buffer), mobile phase B conducted with acetonitrile, mobile phase C conducted with water and a gradient elution, a flow of 1 mL/min, an injection volume of 5 μL, and an analysis time of 35 min.

### 2.5. Data Modelling

In prior analysis, the data were cantered and standardized. To check the presence of outliers or extreme values, we visualized the AA distributions as density plots and quantile–quantile plots. The normal distribution of the AA data was checked using the Shapiro–Wilk test. To check whether the distribution of each AA differed between investigated groups, we performed either a Kruskal–Wallis test (for non-normal distribution) or a *t*-test (for normally distributed data). To check the presence of linear correlations between the amino acids, a Pearson correlation coefficient was calculated for each amino acid against each other. The relationship between the categorical variables and the disease status was checked using the Fisher test.

#### 2.5.1. Random Forest Algorithm

The random forest (RF) model was used to evaluate the impact of amino acids on classification between groups. The RF algorithm is a machine learning-based algorithm constructed from decision trees trained using bootstrap aggregation, where each new tree is fit from a bootstrap sample of the training observations. The out-of-bag (OOB) error is the average error for each, calculated using predictions from the trees that not contained in their respective bootstrap samples. The model was built for 22 variables, which we hypothesized would have an impact on classification between both groups.

#### 2.5.2. Missing Values and Unbalance in the Data Structure

As the data (50 controls and 73 cases) had missing values and were unbalanced, we first imputed the missing values. The imputation technique was specified via the impute function, relative to a feature class. Next, we split the data into training and test sets (7:3) with further application of the random over-sampling examples (ROSE) algorithm to balance it [21].

#### 2.5.3. Model Development and Variable Importance

The RF model was fitted using the training data, with the setting for the optimal number of trees and variables giving the lowest error rate on the training set. After training the RF model, we assessed the variable importance to address the question of which variables were the most important in building the model and had a significant impact on the outcome. The variable importance was calculated based on the Gini index by calculating each feature’s importance as the sum over the number of splits across all tress that included the feature, proportional to the number of samples it split.

#### 2.5.4. Model Performance

The model performance was checked on the validation set using such metrics as accuracy, sensitivity, specificity, and area under the curve (AUC). The variables based on the Gini index were further included in the logistic regression model. The inference on their impact was based on the calculation of the odds ratio (OR), a 95% confidence interval and the *p*-value.

All analyses were conducted in R (7. R Core Team, 2014), and figures were produced using the package ggplot2.

## 3. Results

Comparison: Elderly subjects with no dementia as the control group (*n* = 50) versus elderly patients with dementia (*n* = 73).

**Figure 1:**
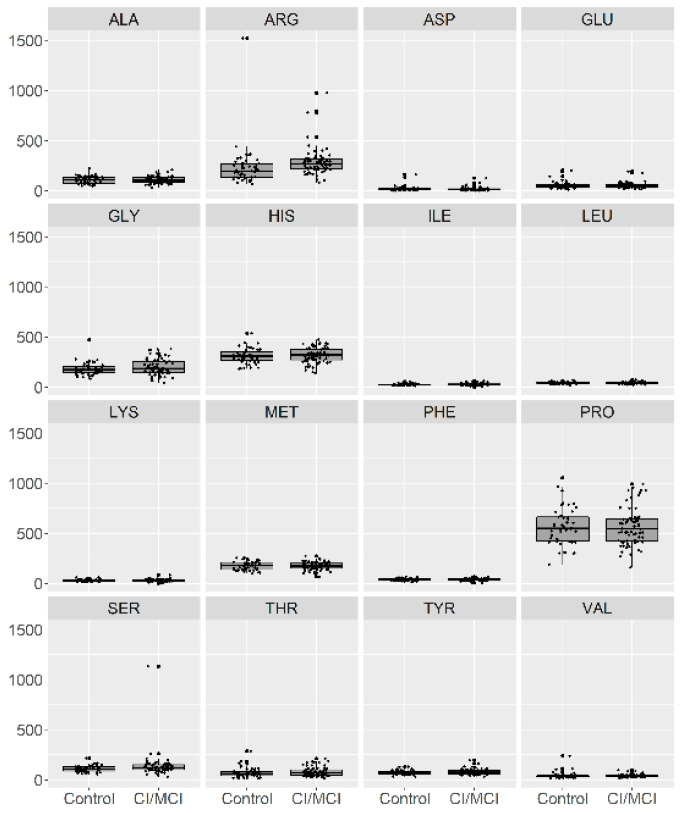
Visualization of the raw data for 16 amino acids in patients with no cognitive impairment or with Mild Cognitive Impairment (MCI) (depicted as 0) and with mild or moderate dementia (depicted as 1).

**Figure 2:**
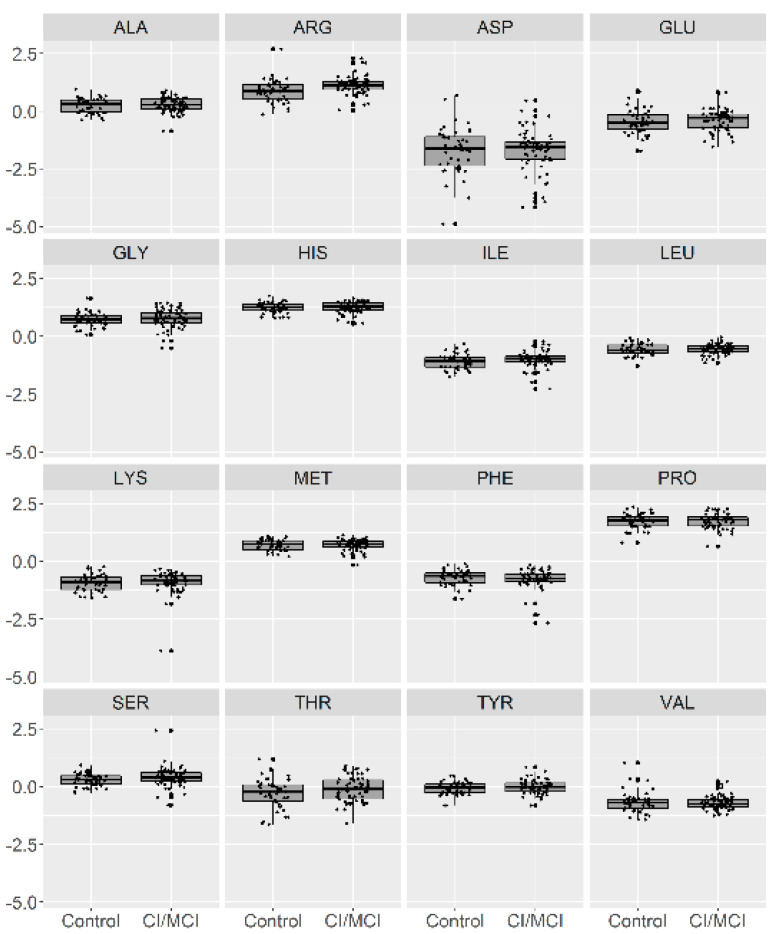
Visualization of the centered and standardized data for 16 amino acids in patients with no CI or with MCI (depicted as 0) and with mild or moderate dementia (depicted as 1).

**Figure 3:**
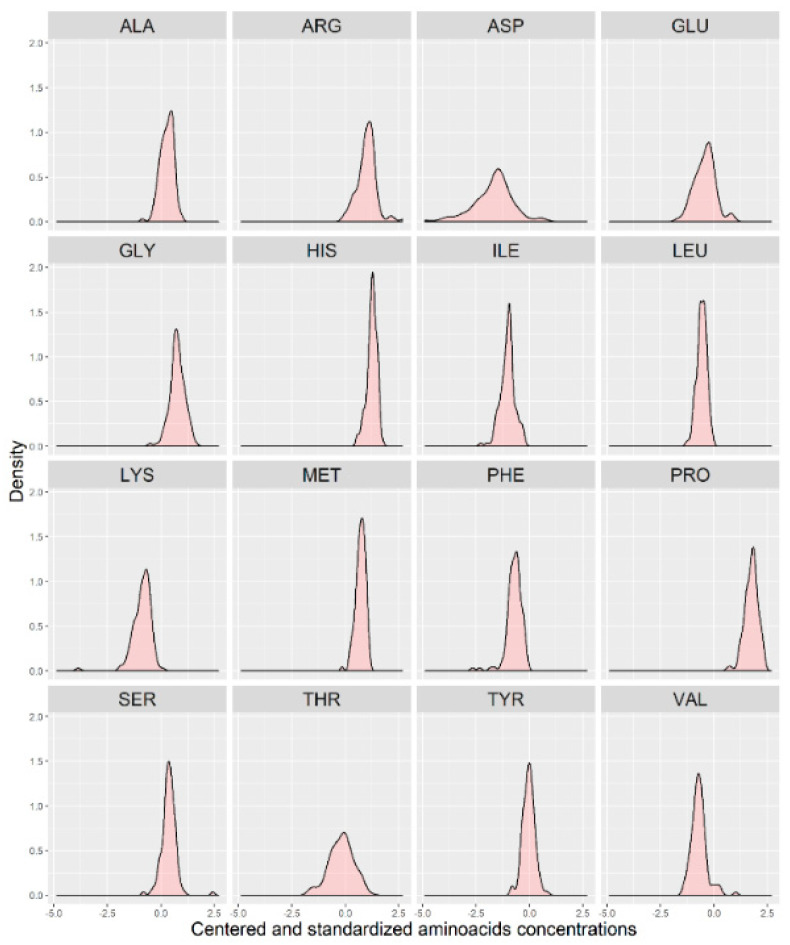
Distribution of the centered and standardized amino acids in a group of 123 individuals (with no separation between the two groups).

In Figure 1, we present a visualization of the raw data for each AA (asparagine (ASP), serine (SER), glutamine (GLU), glycine (GLY), histidine (HIS), arginine (ARG), threonine (THR), alanine (ALA), proline (PRO), tyrosine (TYR), valine (VAL), methionine (MET), lysine (LYS), isoleucine (ILE), leucine (LEU), and phenylalanine (PHE)) for the two groups. To maintain the same scale for each AA and for data analysis purposes, we centered and standardized the raw values. In Figure 2, we present a visualization of the centered and standardized data for each AA for the two groups. From a visual inspection, we might observe a slight difference in the ARG mean between both groups. Not all amino acid concentrations were normally distributed. Under such a scenario, we calculated the average and SD for each distribution, but only for those AAs with normal distributions (Figure 3, Table 2).

**Figure 4:**
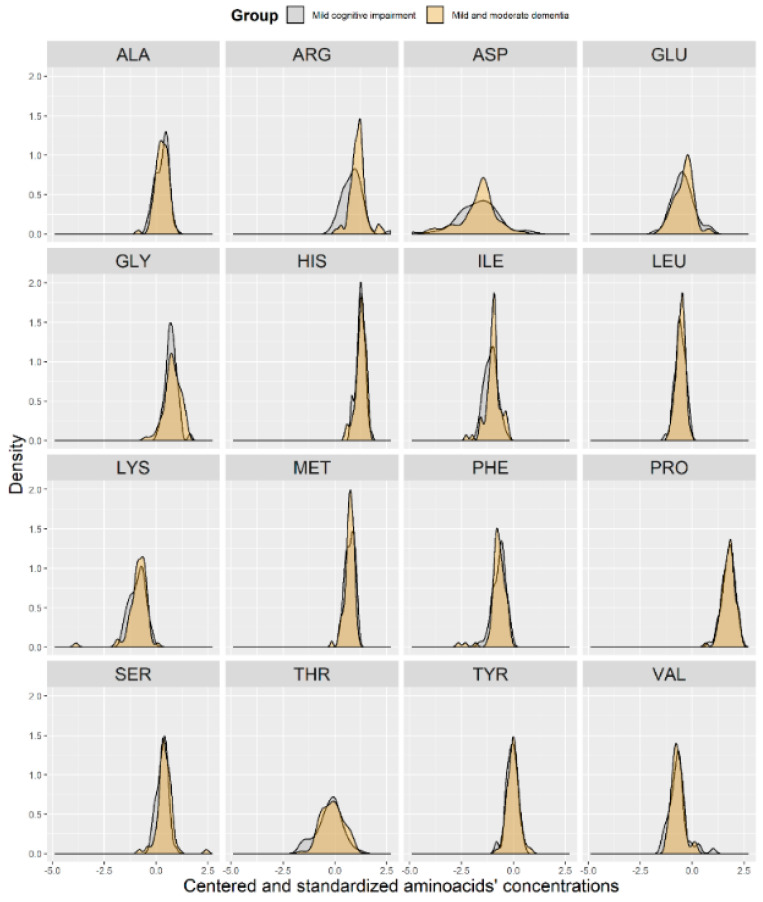
Distribution of the centered and standardized amino acid concentrations between the two groups (controls (grey) versus cases (yellow)).

To investigate whether AA distributions were homogeneous and whether the AA concentration data originated from two or more different overlapping distributions (e.g., groups), we provided the distribution of each AA in the control (*n* = 50) and case (*n* = 73) groups (Figure 4). As shown in Figure 4, separation between the two groups might be expected for ARG. At α = 0.05, the distribution between both groups significantly differed for SER (*p* = 0.042), ARG (*p* = 0.0003), and ILE (*p* = 0.035).

**Figure 5:**
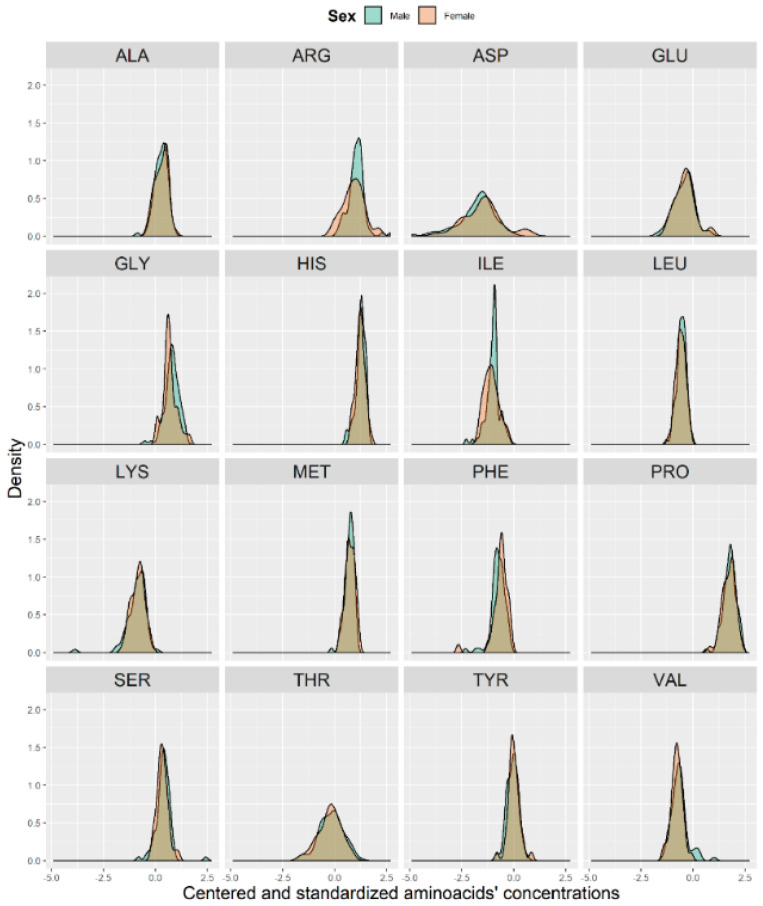
Histograms of the centered and standardized amino acid concentrations between males (blue) and females (pink).

Additionally, we checked the distribution of AA concentrations between sexes (Figure 5). As shown in Figure 5, the distribution between sexes seemed to be almost identical.

The authors analyzed the influence of age on amino acid concentrations to check whether there was any trend (e.g., higher or lower concentration with age). Neither a positive nor a negative trend with age was observed in the data.

We further investigated the relationship between alcohol consumption, smoking status, concomitant diseases, drugs intake, and diet and the occurrence of cognitive impairment (using the Fisher exact test). No relationship between alcohol intake, smoking status, and drug intake and cognitive impairment was found. Only the relationship with diabetes (*p* = 0.009) was found to be significant at α = 0.05.

**Figure 6:**
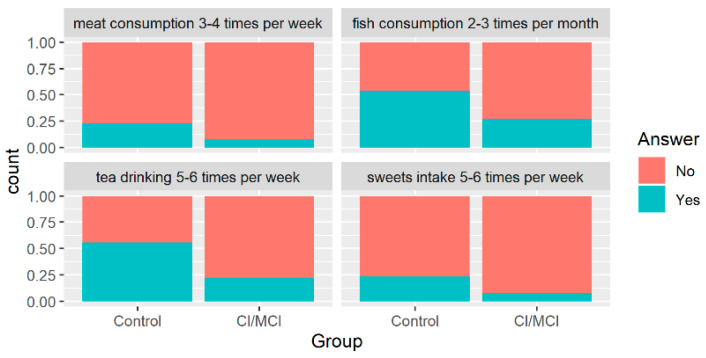
Distribution of patients’ groups with regard to their classification (x-axis) and the proportions for categorical variables which appeared to be significant (α = 0.05) in Fisher’s test.

However, we found a significant difference between the presence of mild-to-moderate dementia and the frequency of meat consumption (3 or 4 times per week, *p* = 0.007), tea drinking (5 or 6 times per week, *p* = 0.0008), fish consumption (2–3 times per month, *p* = 0.044), and sweets intake (5–6 times per week, *p* = 0.044). The visual representation of the above-mentioned significant differences is presented in Figure 6. However, these results should be interpreted with caution as the investigated group was small, and a large disproportion between the compared groups was present.

**Figure 7:**
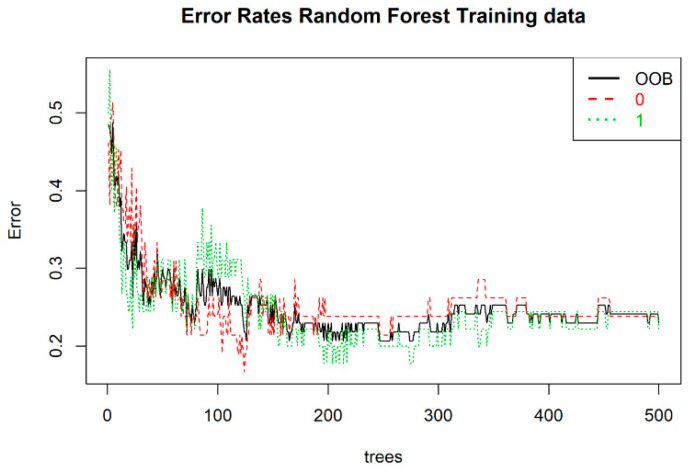
Error rate for the random forest training set. On the x- and y-axis, one can observe the number of trees and the error rate, respectively. The out-of-bag (OOB) score for the model is presented as a black solid line; 0 and 1 represent the control and case groups, respectively.

The random forest model was built for 22 variables, which we hypothesized had an impact on the splitting between both groups. The OOB error rate was the lowest with 150 trees (Figure 7). The optimal number of variables was set at three.

The missing values were imputed using the medians or modes, depending on the variable type. The available data were highly unbalanced (referring to classification problems where we had unequal instances for different classes). As a consequence, an unbalanced dataset would bias the prediction model towards the more common class. We artificially balanced the samples according to a smoothed bootstrap approach and allowed for aiding both the phases of estimation and accuracy evaluation of a binary classifier in the presence of a rare class [21].

**Figure 8:**
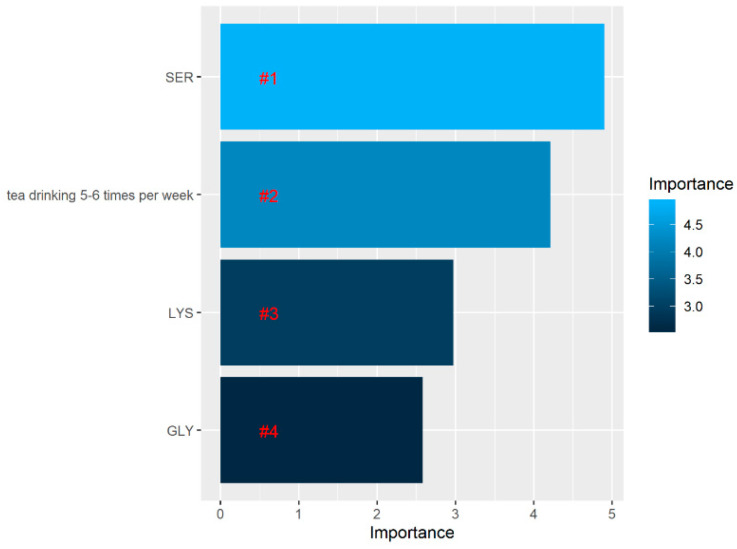
Variable importance assessing the relative importance of individual predictors in the random forest model. According to the legend on the right, the lighter the color, the higher the contribution of the variable to the classification between patients with and without impairment.

In order to select the most relevant variables contributing to patient classification, after developing the RF model, we assessed the contribution of each variable to the classification between both groups. The RF algorithm selected the top four variables (Figure 8).

The accuracy, sensitivity, specificity, and AUC of the RF model is presented in Table 3.

Based on 16 amino acid concentrations, the greatest separation between both groups (healthy elderly subjects with no dementia diagnosed and patients with mild or moderate dementia) was served by arginine, serine, and isoleucine. Significantly increased concentrations of serine, isoleucine, and arginine were present in patients with dementia compared with the elderly subjects without dementia. In the RF model, serine appeared to be the most important, and in the logistic regression model, this variable appeared to be significant.

Based on the *p*-values, only the SER concentration and drinking tea 5–6 times per week appeared to be significant at α = 0.05. An OR > 1 denotes a risk of mild-to-moderate dementia, while an OR < 1 represents a protective effect. A one-unit increase in the SER concentration led to a 1.51-fold increase in the odds of the presence of mild-to-moderate dementia. Tea drinking was negatively correlated with having mild-to-moderate dementia. There was a 74 percent reduction in the odds of having mild-to-moderate dementia for those with a tea drinking frequency of 5–6 times per week compared with patients with no CI or MCI (Table 4).

To further study the nature of amino acids, we checked correlations between the amino acids against each other. Since the distribution for each AA resembled a normal distribution and AA originate from similar pathways, we may expect a linear dependence between them. Thus, we used a Pearson correlation, which measures the linear dependence between variables.

**Figure 9:**
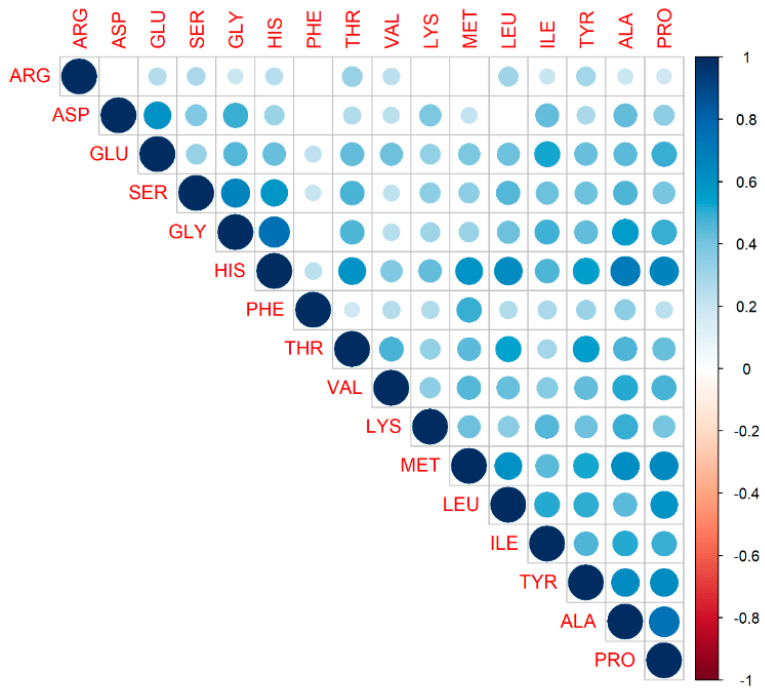
Correlation matrix demonstrating the degree of linear relationship between amino acids. The variables are ordered according to the value of the Pearson correlation coefficient. The lighter the color, the lesser the coefficient value between two variables. Only significant correlations are depicted; insignificant correlations are leaved blank. No negative correlation between amino acids was observed.

In Figure 9, we present a correlation matrix demonstrating the degree of linear relationship between the AAs. Only positive relationships between amino acids were observed, which was expected, taking into account that AA origins might be driven by a similar mechanism. As observed in this figure, they carried essentially the same information.

## 4. Discussion

Metabolomic approaches represent a promising, potent, and comprehensive tool for the identification of a wide range of biochemical changes that are associated with neurodegenerative diseases and their treatments. Recent scientific evidence associates metabolic dysfunctions with neurodegenerative development and the severity thereof. However, despite significant technological and research progress, there is as of yet no unequivocal evidence pointing to accurate biomarkers of the disease in human models. Several researchers support the assumption that AD is a systemic disorder characterized by mitochondrial dysfunction, glucose metabolism, and altered amino acid metabolism [6,22]. The results of this study indicate that the serum levels of three amino acids changed significantly in patients with the initial stages of dementia, in relation to elderly subjects without dementia.

In particular, we observed differences in concentrations for SER (significantly increased in patients with dementia compared with the control group), ARG (significantly increased in patients with dementia compared with the control group), and ILE (significantly increased in patients with dementia compared with the control group), which were supported by visual inspection. In the RF model, serine appeared to be the most important. Based on the *p*-values, a one-unit increase in SER concentration led to a 1.51-fold increase in the odds of the presence of dementia.

It is interesting to note that D-serine, formed from L-serine by serine racemase, is the physiologic main co-agonist at the N-methyl-D-aspartate receptors (NMDARs) subtype in the frontal brain areas. An association between the incorrect activation of the glutamate receptors of the NMDARs, synapse dysfunction, and neurotoxicity in AD was reported [23,24,25,26,27,28,29,30,31,32]. Researchers confirmed the serine implication in NMDAR-mediated neurotoxicity [23,24,25,26,27,28,29,30,31,32,33]. Previously, in vitro studies showed an increased release of D-serine from neuronal and glial cells and NMDAR activation under injury in AD model systems [23,34,35].

Madeira et al. measured the serine levels in post-mortem samples from subjects without dementia and AD patients, and they reported that D-serine levels were significantly higher in the CSF of AD patients than in the control group. They showed that increased levels of brain and CSF D-serine are associated with AD. Furthermore, they indicated the significant correlation between increased D-serine in CSF and poorer cognitive performance. They suggested that D-serine could be a novel AD biomarker [23]. The present findings seem to be consistent with other research, which found increased D-serine levels in the CSF of AD patients compared with the control group [36].

As mentioned in the literature review, increased serine levels in the CSF and brain tissue samples from patients with advanced neurodegenerative diseases were previously reported. However, in reviewing the literature, no data was found for serine measured in non-invasive material from patients with early stages of neurodegenerative diseases. In this study, we showed the increase level of serine in the initial stage of dementia (mild and moderate dementia) with a relatively easy method involving a non-invasive material sampling technique. We acknowledge that this is an introductory pilot study and, thus, a larger study on greater samples is warranted to extend the validity of our results. Isoleucine is one of the essential branched-chain amino acids (BCAAs). Larsson et al. noticed that the uptake of BCAAs into the brain occurs at the blood–brain barrier via a competitive transport carrier that they share with tryptophan. Tryptophan is the precursor of serotonin and other large, neutral amino acids. A long-term increase in the levels of branched-chain amino acids could lead to reduced levels of brain tryptophan, as well as the decreased synthesis of neuronal serotonin and serotonergic signaling. Finally, this may increase the risk of the development of neurodegenerative diseases such as AD [37,38]. In 2017, Larsson and a coworker demonstrated that a genetic predisposition to higher levels of isoleucine in plasma is positively associated with the occurrence of AD. They suggest that lifelong increased levels of isoleucine may raise the risk of AD [37]. The results of our study seem to be consistent with previous research and suggest that isoleucine increases in patients with dementia.

Finally, we observed the significant increase in concentration of arginine in patients with dementia (from mild to moderate) compared with the healthy control group. This finding is in agreement with Corso et al. and their findings, which showed the trend toward increased arginine concentration in dementia caused by AD [15]. Furthermore, our results are consistent with those of Olazaran, who found the trend of arginine growth along the continuum from the control group (healthy subjects) to mild cognitive impairment and to AD [39].

Fonteh et al. showed that an increase in the concentration of arginine in urine and plasma, accompanied by a decreased in the arginine concentration in CSF, is evidence of a change in nitrogen detoxification in AD [18]. Furthermore, a number of recent studies have found the implications of alterations in arginine metabolism in the pathogenesis of neurodegenerative disorders. In 2014, Liu et al. reported that arginine metabolism definitely changed in diverse regions of the brain in AD and emphasized that further research should be done to understand its role in the pathogenesis of the disease. It is important to note that arginine is a precursor for nitric oxide and polyamines. Both are essential modulators of neuronal physiology and are thought to be involved in the pathogenesis of neurodegenerative diseases such as dementia [40]. Nitric oxide is produced from L-arginine, and it has numerous physiological functions such as learning, memory, noradrenaline and dopamine release, and regulation of the cerebrovascular system. It also takes part in some pathologies, such as schizophrenia, stroke, cerebral ischemia, and Huntington’s disease [41].

In 2015, Graham et al. published a paper in which they described the analysis of human plasma and how it indicated differentially affected polyamine and L-arginine metabolisms in mild cognitive impairment subjects converting to AD. Researchers found that the L-arginine metabolism and polyamine metabolism were interlinked, sharing common metabolite intermediates. They are connected via the urea cycle through the enzyme arginase, which converts L-arginine to urea and L-ornithine. Researchers indicated that enzyme arginase appeared to control the proliferation and apoptosis of neural cells. Their results demonstrated that, in MCI and in AD, there was a significant increase in L-arginine which was coupled with a decrease in L-ornithine. It has been suggested that this disturbance in the arginase pathway may potentially reflect alterations in neurogenesis [42,43,44]. These results and their interpretations, based on the literature, suggest that the metabolisms of some amino acids seem to be changed in patients with dementia. However, at this point, we highlight that the main limitation of this study relates to small and unbalanced sample sizes. Therefore, the obtained results should be confirmed with a larger sample size and should be interpreted with caution to fully confirm their validity. Furthermore, amino acid modifications could be a consequence and not a cause of AD, and they also should be considered as limitations. Factors affecting plasma amino acid concentrations should also be considered. Therefore, our findings need further validation, especially regarding potential alterations in energy metabolism in patients with dementia. It seems valuable for future studies to include different biological materials (tissue and fluid samples) from one patient for a more comprehensive analysis. Long-term studies to measure changes in amino acid concentrations versus dementia progression (from mild to advanced stages) would also allow for a more thorough analysis.

## 5. Conclusions

We conclude that amino acid profiling might be helpful in better understanding biochemical and metabolic changes related to the pathogenesis and progression of dementia. In the present study, we discovered significant changes in serine, isoleucine, and arginine concentrations in patients with dementia compared with elderly subjects without dementia. These changes may each play a different role in the disease, and this highlights the multifactorial, heterogenous, and complex nature of this disease.

While this information is very useful, it is not sufficient by itself for good understanding of the pathological changes associated with dementia. Considering the multifactorial, heterogenous, and complex nature of this disease, validation with a greater study sample in further research is required.

## Figures and Tables

**Table 1:** Demographic and clinical characteristics of the study groups.

	Control Group (*n* = 50)	Case Group (*n* = 73)	*p*-Value
**Mean age ± SD, years**	77.09 ± 7.08	81.89 ± 6.38	
**Gender, *n* (%)**			*P* = 0.014
Male	23 (46)	17 (23.3)	
Female	27 (54)	56 (76.7)	
**Mean BMI ± SD**	28.01 ± 5.37	25.79 ± 6.34	
**Drugs, *n* (%)**			*P* = 0.664
Painkillers	16 (32)	22 (30.1)	
Duretics	15 (30)	13 (17.8)	
Anti-coagulants	13 (26)	18 (24.7)	
Anticancer medications	0 (0)	1 (1.4)	
Mineral supplements	1 (2)	1 (1.4)	
Thyroid medications	5 (10)	10 (13.7)	
Folic acid	2 (4)	6 (8.2)	
Vitamin D3	5 (10)	13 (17.8)	
**Smoking, *n* (%)**			*P* = 0.862
Non-smoker	39 (78)	55 (75.3)	
Former smoker	4 (8)	6 (8.2)	
Occasional smoker	0 (0)	0 (0)	
Active smoker	0 (0)	2 (2.7)	
Passive smoker	0 (0)	0 (0)	
**Medical history, *n* (%)**			*P* = 0.284
Hypertension	35 (70)	52 (71)	
Diabetes	7 (14)	26 (35)	
Kidney disease	2 (4)	0 (0)	
Liver disease	0 (0)	1 (1.4)	
Thyroid disease	7 (14)	13 (17.8)	
Cancer	4 (8)	10 (13.7)	

**Table 2:** The mean (±SD) for each metabolite signal distribution.

Compound Name	Mean (± SD)
ALA	0.27 (± 0.30)
GLY	0.75 (± 0.35)
HIS	1.24 (± 0.23)
LEU	−0.56 (± 0.22)
MET	0.71 (± 0.22)
PRO	1.74 (± 0.30)
TYR	−0.04 (± 0.27)

**Table 3:** Performance of the random forest model, assessed by different metrics.

	Random Forest Model
**Accuracy**	0.72 (0.47–0.84)
**Sensitivity**	0.64 (0.38–0.83)
**Specificity**	0.78 (0.51–0.85)
**AUC**	0.74 (0.57–0.88)

**Table 4:** Logistic regression estimates with the odds ratio (OR), 95% lower confidence intervals (LCIs), and 95% upper confidence intervals (UCIs) on the whole data set of 123 patients.

Variable	Estimate	OR [Exp(Estimate)]	*p*-Value
SER	1.51	4.53 (1.48–13.87)	0.008
GLY	0.38	1.46 (0.58–3.69)	0.417
LYS	0.17	1.19 (0.61–2.33)	0.6
Tea drinking 5–6 times/week	−1.33	0.26 (0.12–0.53)	0.0002
